# Exploring breast cancer associated-gene panel for next-generation sequencing and identifying new, pathogenic variants in breast cancer from western China

**DOI:** 10.7150/jca.101911

**Published:** 2025-01-13

**Authors:** Jingliang Cheng, Binghui Song, Chunli Wei, Lianmei Zhang, Xiaoyan Liu, Lisha Yang, Singkome Tima, Sawitree Chiampanichayakul, Xiuli Xiao, Songyot Anuchapreeda, Junjiang Fu

**Affiliations:** 1 Key Laboratory of Epigenetics and Oncology, The Research Center for Preclinical Medicine, Southwest Medical University, Luzhou 646000, Sichuan Province, China.; 2 Department of Medical Technology, Faculty of Associated Medical Sciences, Chiang Mai University, Chiang Mai 50200, Thailand.; 3 Department of Pathology, The Affiliated Huaian No. 1 People's Hospital of Nanjing Medical University, Huai'an 223300, Jiangsu Province, China.; 4 Research Center of Pharmaceutical Nanotechnology, Chiang Mai University, Chiang Mai, 50200, Thailand.; 5 Department of Pathology, the Affiliated Hospital, Southwest Medical University, Luzhou 646000, Sichuan Province, China.

**Keywords:** breast cancer, gene panel, next-generation sequencing (NGS), pathogenic variants, gene diagnosis, genetic counseling

## Abstract

Breast cancer (BC) is the most frequently diagnosed and the leading cause of cancer-related deaths among women worldwide. It is crucial to develop a cost-effective BC genetic panel for detection and diagnosis. In this study, tissue samples from 52 BC patients and peripheral blood samples from 18 healthy volunteers were collected in western China, followed by gDNA extraction. H&E and IHC analysis were employed to detect the expression of invasive BC tissues. We analyzed data using public databases such as COSMIC/ClinVar/HGMD along with our own previously published data and queried commercial BC panels to select high-risk genes. Using Illumina DesignStudio, gene panel primers consisting of 13 genes were designed with 696 primer pairs. The specificity of all primers was validated through common PCR assays. Once the gene panel was completed, multiple polymerase chain reactions (MPCR) were performed using the designed panel primers. The resulting MPCR products were purified to enrich them as library templates. Subsequently, after passing quality tests for library integrity assessment, Next-generation sequencing (NGS) was conducted. Through bioinformatics analysis of the NGS data, 4,571 variants were identified in the annotation files from 52 samples, classified into different types. Among these variants, 358 (approximately 7.8%) were newly discovered and distributed across 11 genes in 52 patients without in the ExAC database. The *KMT2C* gene exhibited the highest frequency of variants, presenting in 83.0% of 52 patient samples. Variants in *BRCA2* (71%), *BRCA1* (48%), *PALB2* (40%), *PIK3CA* (23%), and *RNF40* (21%) genes were found in over 20% of patients. Additionally, variants were observed in the *AKT1* (12%), *ERBB2* (10%), *ESR1* (8%), *TWIST1* (8%), and *PIK3R1* (4%) genes. Further analysis using PolyPhen-2, SIFT, CADD, and Mutation Taster tools analysis showed that out of these new variants, 49 (49/358) had potential pathogenic effects on protein functions and structure across 52 patients. Consequently, a high-risk gene panel has been preliminarily established for early detection/diagnosis that will contribute to earlier prevention and treatment strategies for individuals with BC, particularly those residing in developing or underdeveloped countries. The identification of novel pathogenic variants within our cohort not only expands knowledge regarding genetic diagnosis applications for BC patients but also facilitates genetic counseling services for affected individuals and their families.

## 1. Introduction

Cancer is a prevailing global healthy issue, particularly in developing or undeveloped countries or regions. According to the GLOBOCAN 2022 data 1, 2, breast cancer (BC) has been reported as the second most frequently diagnosed and the fourth leading cause of death among all cancers worldwide. Especially, BC is the most common cancer and the main cause of cancer death among women worldwide. In China, there were approximately 357,200 new cases of BC reported in females in 2022 3, accounting for 15.47% of new female cases globally with around 75,000 deaths. The increasing incidence and mortality rates of BC patients have made this disease a significant threat to the health of Chinese women. Advancements in clinical diagnosis and treatment have significantly improved BC survival rates, however, there remains a gap between the five-year survival rate of BC patients in China (82.0%) and that observed in developed countries like the United States (90.9%) 4-6. The discrepancy can be attributed to delayed diagnoses among many BC patients who are already at intermediate or advanced stages with tumor metastasis upon initial detection. Consequently, surgical interventions may occur too late resulting in high post-operative recurrence rates and shorter survival times. To address the challenge posed by rapidly increasing incidence and mortality rates among BC patients, early detection/diagnosis, prevention strategies, and timely treatment must be prioritized 5.

Precision medicine is an effective approach based on scientific understanding of individual differences arising from genome variation, environment, and lifestyle. It involves precise genetic diagnosis to achieve personalized therapy, and early prevention 7, 8. In developed Western countries, databases such as ClinVar, the Catalogue of Somatic Mutations in Cancer (COSMIC), next-generation sequencing (NGS) technology, and bioinformatics analysis, are utilized for genetic diagnosis in BC patients. Commercial gene panels have already been developed to cater to their needs 5, 6, 9, 10. However, in China-a developing country with certain underdeveloped regions like western China-factors such as living habits, educational level, and medical conditions (particularly scientific and technological advancements), contribute to a large number of BC patients being diagnosed at advanced stages with poor prognosis 2, 5, 6, 9-11. BC has become a prevalent malignant tumor significantly impacting the physical and mental health of women while posing a threat to their lives. The primary cause of death among BC patients lies not in the growth of primary tumors but rather in metastasis and recurrence. Therefore, it is imperative to develop a cost-effective gene panel that can effectively facilitate early prevention, detection, and diagnosis.

The etiology and pathogenesis of BC are multifactorial and complex, with genetic factors playing an influential role in approximately 5%-10% of cases, while the majority of cases (90%-95%) are sporadic and caused by somatic mutations. Somatic mutations are not inherited, and the primary method employed in this study involved in collecting cancer tissue for comparison with normal tissue or blood samples. The COSMIC database serves a comprehensive resource for exploring the impacts of somatic mutations on different cancers. Notably, *BRCA1* and *BRCA2* genes are the most common disease genes associated with hereditary BC due to their high-frequency germline mutation. Currently, variant interpretation guidelines provided by the American College of Medical Genetics and Genomics/Association for Molecular Pathology (ACMG/AMP) solely focus on germline variants. However, advancements in DNA sequencing techniques such as NGS have facilitated the identification of other BC susceptibility genes including various variants of *TP53*, *PIK3CA*, *PALB2*, and *PTEN* genes. Furthermore, rare genes have been reported to increase the risk of developing metastatic BC 10, 12-14. This study aims to establish a high-risk gene panel for the diagnosis BC in western China to enhance knowledge and information pertaining to the clinical application of genetic diagnosis among BC patients. These findings can provide valuable insights for genetic counseling related to both BC patients and their family pedigrees.

## 2. Materials and Methods

### 2.1 Sample collection and DNA extraction

**Figure 1:**
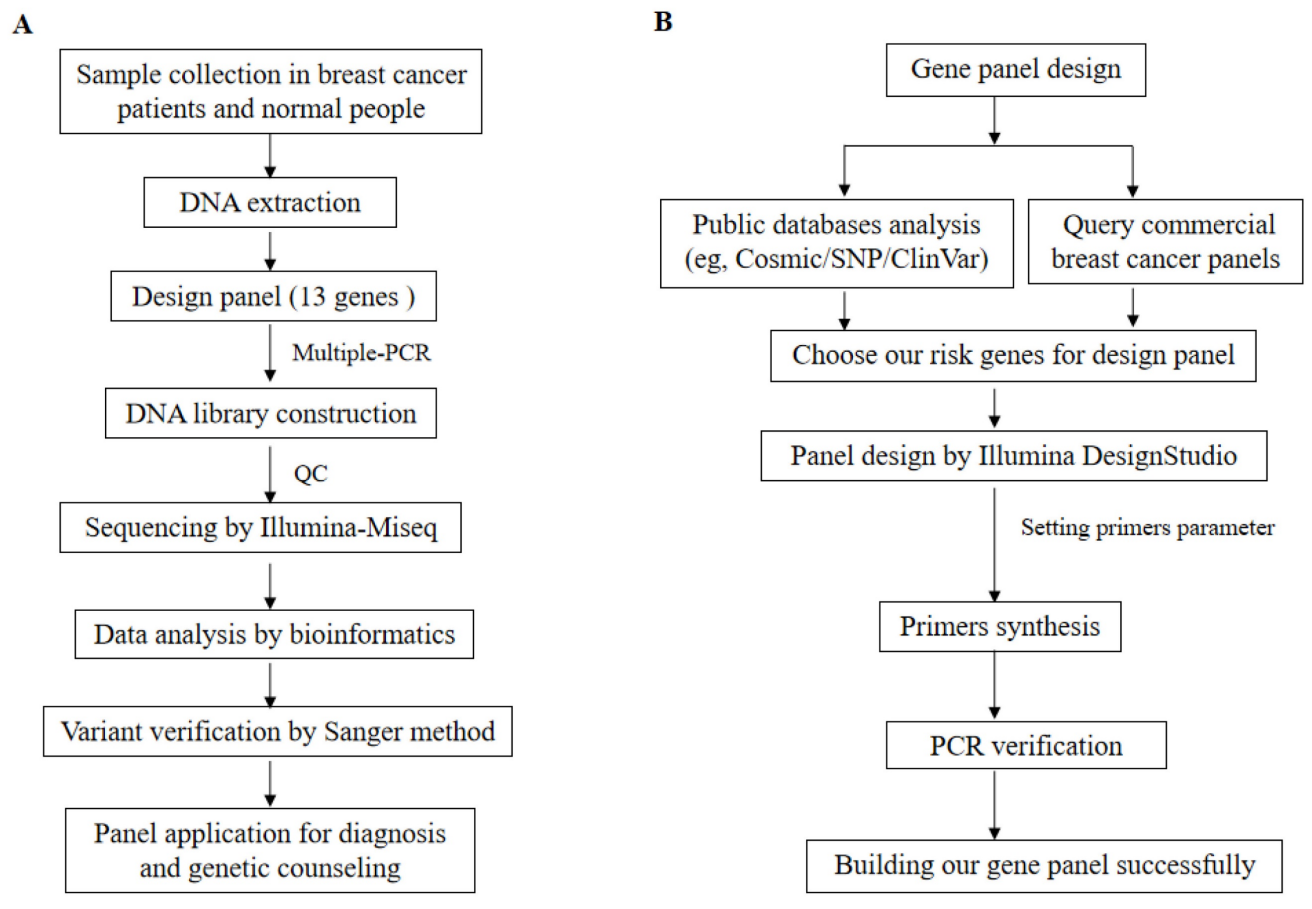
The workflow of our study and gene panel design is as follows. A. Study workflow - firstly, we collected BC tissue from patients and blood samples from normal individuals. Subsequently, DNA was extracted, followed by use of 13 genes in the design panel. Multiple-PCR was completed and the product was purified. Then, a DNA library was constructed, quality control checks were performed using Agilent 5200, and sequencing was carried out using the Illumina Miseq platform. Thirdly, bioinformatics analysis of sequencing data obtained all variants which validated using the Sanger method before applying for diagnosis and genetic counseling through the panel. B. Gene panel design workflow - firstly public databases (e.g., Cosmic/SNP/ClinVar) were analyzed along with commercial BC panels to choose risk genes for designing our gene panel. Secondly, Illumina DesignStudio designed gene panels while setting primer parameters. Thirdly, synthesized primers underwent PCR validation to ensure specificity. Finally, the successful construction of our gene panel.

This study was approved in advance by the Ethics Committee of Southwest Medical University and was conducted in accordance with the principles outlined in the Declaration of Helsinki (2013 version). Tissue samples were recruited from 52 patients with BC, along with blood samples from 18 healthy donors from different families. The patient was diagnosed with BC through pathological examination, and the BC tissue of the patients collected through clinical surgery. Normal people have no disease after clinical examination, and blood samples of normal people are collected through clinical physical examination. Clinical examination and family information, including age, sex, tumor sizes, tumor stage, tumor metastasis, pathological diagnosis, and family history were recorded for BC patients. The expression of Her-2/ER/PR patient pathological samples was utilized for BC pathology reporting across various subtypes. Tumor size and metastasis served as grading systems to determine patient status. Subsequently, DNA extraction was performed using a standard phenol/chloroform method 15 on both BC tissues and normal blood samples obtained from patients and healthy donors, respectively. The workflow of this study is illustrated in Figure 1A.

### 2.2 Hematoxylin-Eosin staining and immunohistochemistry

Patients with BC tissues were fixed in 10% formalin for one day, embedded in paraffin, and sliced every 5 µm. After dewaxing in xylene and dehydrating, the slides were stained with hematoxylin and eosin (H&E) before being probed by immunohistochemistry (IHC). The H&E and IHC methods were performed as previously described protocols 16-18. The BRCA1 (cat #: ZM-0347) and TP53 (cat #: ZM-0408) antibodies for IHC were purchased from Beijing Zhong Shan-Golden Bridge Biological Technology Co., Ltd., CN. Slides with a thickness of 5 µm underwent antigen retrieval through incubation in 10 µM sodium citrate buffer at 95°C for three times, 12 min each. Samples were then blocked using a solution containing 5% BSA before primary antibodies for BRCA1 and TP53 (diluted at a ratio of 1:100) were applied overnight. Appropriate biotin-conjugated secondary antibodies (cat #: SP-9000, ZSGB-Bio, CN) were used to incubate the samples at room temperature for an hour before visualization was achieved via streptavidin-conjugated horseradish peroxidase (HRP) and 3,3-diaminobenzidine (DAB) (cat #: ZLI-9017, ZSGB-Bio, CN). Finally, the stained slides underwent retaining with hematoxylin prior to dehydration, mounting, and analysis.

### 2.3 Design gene panel

To design the gene panel, we initially conducted an analysis using public databases such as COSMIC/ClinVar/HGMD. Specifically, a comprehensive analysis of the COSMIC database was performed as previously described 19. Subsequently, the top twenty genes were identified with a mutation frequency in BC and searched for specific inhibitors to target these genes through literature research 7, 12, 14, 20-32. Additionally, considering our previous studies and current investigation on BC genes to select high-risk genes specific to the Chinese population in our region 16, 33-35, they were included in the gene panel. Furthermore, to tailor our panel specifically for Chinese individuals at high risk of BC development, commercially available BC panels were compared from Western developed countries 6, 10, 32, 36-38. Thirteen genes (*AKT1, BRCA1*, *BRCA2*, *ESR1*, *ERBB2*, *KMT2C*, *PALB2*, *PIK3CA*, *PIK3R1*, *PTEN*, *TP53*, *RNF40*, and *TWIST1*) were selected for primer design to cover all exons and intron junction sequences. The Illumina DesignStudio was employed to design the primers and set their parameters. The primer set underwent multiple assay steps (AmpliSeq for Illumina Gene) to ensure accuracy before displaying all gene primer information on a webpage table that could be downloaded. Consequently, thirteen genes with corresponding primers pairs totaling up to 696 were synthesized into our designed gene panel. Then, the specificity of primers was assessed by PCR. Finally, the successfully implementation of NGS resulted in the completion of our designed gene panel. The workflow illustrating the process is depicted in Figure 1A &B.

### 2.4 Multiple-PCR and product purified analysis

The multiple-PCR (MPCR) amplification was conducted using genomic DNA (gDNA) as a template in a total volume of 50 µL on a Veriti™ 96-Well Thermal Cycler. The Multiplex PCR Plus Kit for MPCR purchased from QIAGEN, Germany (cat #: 206152) 39 was employed. The components for MPCR were as follows: 25 µL of 2× Multiplex PCR Master Mix*, 5 µL of 10× primer mix, ≤300 ng of template DNA, and RNase-free water to reach a final volume of 50 µL. The cycling protocol for multiplex PCR consisted of an initial denaturation at 95 ºC for 5 min, followed by 35 cycles at 95 ºC for 30 s, then annealing at 60 ºC for 90 s, extension at 72 ºC for 30 s, and final extension at 68 ºC for 10 min. The amplified MPCR products were purified using AMPure XP Beads (Vazyme Biotech Co., Ltd., N411-01).

### 2.5 Construction of gene panel library and NGS analysis

After the successful design of the gene panel, multiplex PCR (MPCR) was performed to amplify our designed panel primers as the library template. The QIAseq 1-Step Amplicon Library kit (QIAGEN, Germany, Cat #: 180412) was utilized for library construction. Subsequently, each sample library was uniquely identified using different adapter sequences. To assess the quality of the libraries, Agilent Fragment Analyzer Systems (Agilent, USA) were employed for library quality control analysis. Each captured sample achieved a depth exceeding 200 × with over 90% coverage. The raw average data size for each sample amounted to 0.7GB, while the sequencing reads exhibited a quality score value around 20, and a base recognition rate surpassing 99%.

NGS was conducted on the Illumina MiSeq platform (Illumina, USA). For bioinformatics analysis of the NGS data, original reads were converted in FASTQ format and aligned to hg19 using BWA aligner version 0.5.9 40. Recalibration and local realignment were performed using Genome Analysis Toolkit (GATK version 1.0.5974) 41, followed by variant calling with Atlas2 toolkit 9. Common polymorphisms with an allele frequency higher than 0.5% were filtered based on several common variant databases. Variant annotation was carried out using ANNOVAR 42 with RefSeq genes as a reference for coordinate mutations. The variant allele frequency of the variants was calculated from sequencing data. Functional predictions of variants were made using SIFT and PolyPhen-2 tools 43. Known pathogenic mutations were searched in various databases such as COSMIC/ClinVar/HGMD/dbsnp/ExAC 14, 44-46. We identified somatic mutations by adding blood samples of healthy individuals to exclude the germline mutations. Loci associated with clinical symptoms underwent screening.

### 2.6 Variant verification by Sanger method

For the validation of variants identified through NGS analysis, gene panel results were analyzed using a designed primers panel. The patient's gDNA was utilized as a template for PCR amplification on a Life Technology (USA) amplification machine, followed by Sanger sequencing analysis on an ABI-3500DX sequencer 15.

### 2.7 Variant classification and bioinformatics analysis

We developed an in-house local knowledge base and a proprietary bioinformatics pipeline to automate the construction of classification types for all identified variants and the variant allele frequency was assessed. The clinical significance of all identified variants was assessed following the standards and guidelines of the American College of Medical Genetics and Genomics by ACMG Laboratory Quality Assurance Committee 12, 47. Minor allele frequencies were analyzed using public databases including the Exome Aggregation Consortium (ExAC) 48, the 5000 Exomes Project 49, the NHLBI Exome Sequencing Project ESP6500 databases 9, etc. Disease-specific variant information was queried from ClinVar 46, Online Mendelian Inheritance in Man (OMIM) 45, COSMIC 44, etc. The variants were analyzed by silico methodology. Variant substitutions' impact on protein structure and function were predicted using SIFT and PolyPhen-2 tools 43. Nucleotide conservation analysis for all variants was performed by phyloP tool 32, 41. An in-house local knowledge base was utilized for classifying, storing, organizing, continuously updating, and upgrading variant information along with lines of evidence. This process ensured reproducibility, rigor, and efficiency in reclassification. Novel variants with our cohort were identified through querying the ExAC database 50, 51. In addition, Combined Annotation Dependent Depletion (CADD) (version 1.7) and Mutation Taster tools were also used to analyze these novel variants 52, 53.

### 2.8 Statistical analyses

The distribution of gene variants among categories was analyzed by assessing alteration in amino acid function. All statistical analyses were conducted using R software version 3.4.4 (R Foundation for Statistical Computing, Vienna, Austria). A statistically significant difference was considered when the adjusted p-value was less than 0.05 54.

## 3. Results

### 3.1 The breast cancer patient recruitment and clinical findings

**Figure 2:**
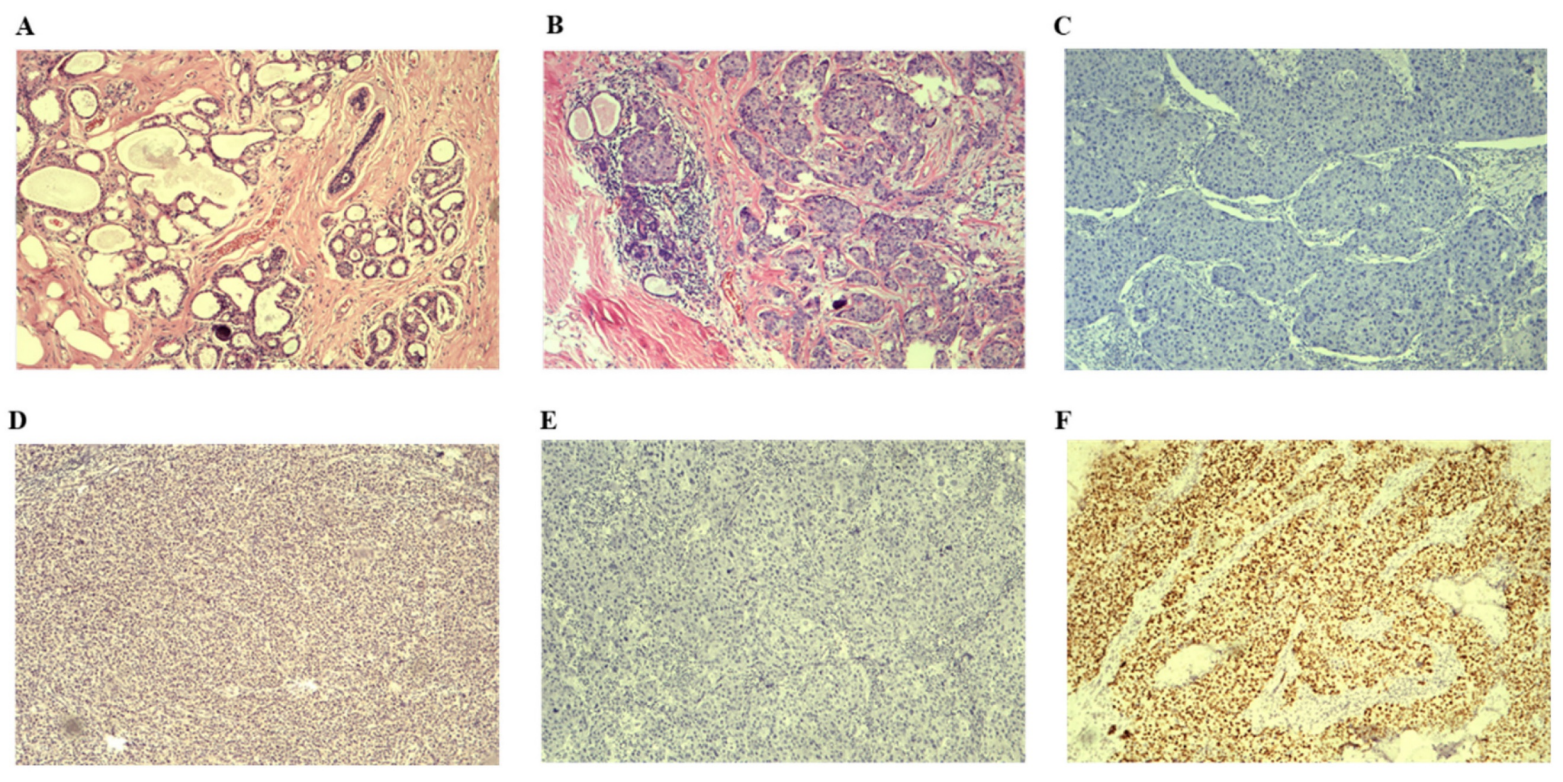
Results of H&E staining images and representative IHC images are presented. A. H&E staining image depicting normal breast tissue is shown. B. H&E staining image illustrating invasive BC tissue is displayed. C. Representative IHC image demonstrating the absence of BRCA1 antibody in invasive BC tissues is provided. D. Representative IHC image displaying the presence of BRCA1 antibody in invasive BC tissues is included. E. Representative IHC image indicating the lack of TP53 antibody in invasive BC tissues is depicted. F Representative IHC image showing the presence of TP53 antibody in invasive BC tissues is exhibited. H&E: Hematoxylin &Eosin; IHC: immunohistochemical staining.

We enrolled 52 patients with BC tissues and 18 healthy blood donors whose DNA passed quality control for the study [Table 1]. Clinical information was collected from all patients, with an average age of diagnosis at 50 years (ranging from 29 to 77 years). The average tumor size was measured at 25 mm (range between 10 and 55 mm). Tumor metastasis was observed in 20 patients (38.5%), predominantly diagnosed as invasive ductal breast carcinomas. Patients were categorized into four subtypes based on the expression of Her-2/ER/PR. Among them, Luminal B subtype had the highest prevalence with 23 patients (44.2%), followed by Luminal A subtype with 14 patients (26.9%). Additionally, 11 patients were classified as Her-2-enriched subtype (21.2% in total), and only four patients were identified as basal-like subtype (7.7%). According to the American Cancer Society (https://www.cancer.org/cancer/types/breast-cancer/about/types-of-breast-cancer.html) and pathological classification of breast cancer (https://mp.weixin.qq.com/s/rP6lUKNQWNP96BuMHeik5A), the breast cancer cases were divided into two types including *in situ* breast cancer and invasive breast cancer. In addition, we categorized invasive breast cancer into early and late stages [Table 1]. Moreover, all BC samples were malignant based on the pathologic diagnosis by two independent pathologists. The pathological examination involved H&E staining and IHC analysis. Representative H&E staining images of normal breast tissue and invasive BC tissue are shown in Figure 2 A&B. Respective IHC images without any antibodies demonstrated negative expression (Figure 2C&E), while strong positive expressions of BRCA1 and TP53 antibodies were observed in invasive BC tissues (Figure 2D&F).

### 3.2 Gene panel design and NGS analysis results

**Figure 3:**
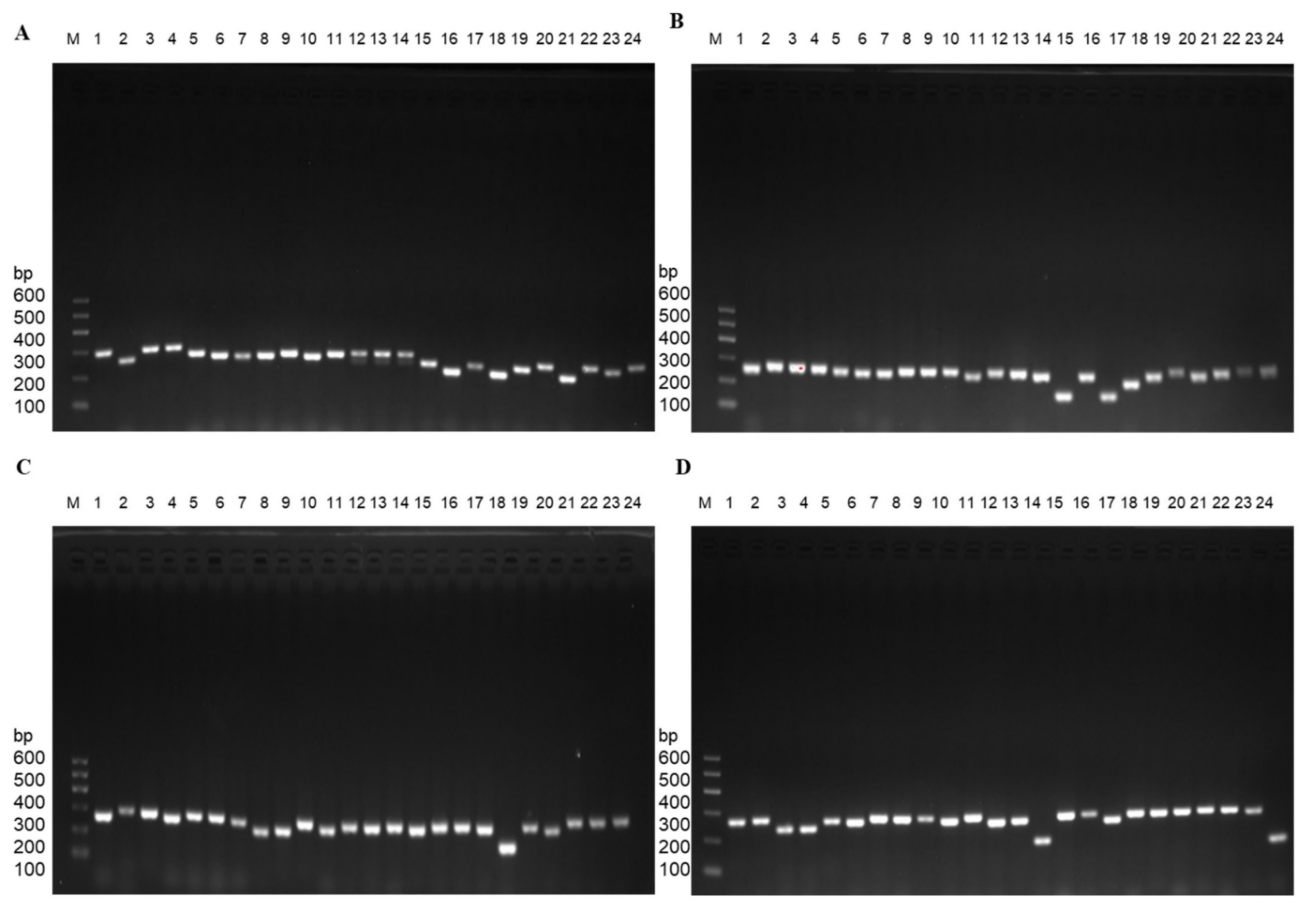
Exemplary PCR results were achieved using gene panel primers. A. *PTEN* gene with partial primers. B. *TP53* gene with total primers. C. *RNF40* gene with partial primers. D. *KMT2C* gene with partial primers.

For the design of gene panel primers, Illumina DesignStudio was utilized and incorporated a total of thirteen genes with 696 primer pairs. The selection of these genes was based on comprehensive analysis using public databases (e.g., COSMIC/ClinVar/HGMD), our previous studies 16, 19, 33-35, as well as a relevant investigation conducted on Chinese breast patients 5, 10, 14, 15, 19, 29, 31, 33, 35, 44-46. Specifically focusing on the *PIK3CA*, *PIK3R1*, *ESR1*, *TWIST1*, *KMT2C*, *PTEN*, *BRCA2*, *AKT1*, *RNF40*, *PALB2*, *ERBB2*, *TP53*, and *BRCA1* genes, the information about chromosome, exons, and primer pairs is shown in Table 2. All the genes frequency is about somatic mutations. Germline mutations were excluded. Subsequently, the primers for the panel were synthesized, followed by PCR verification of their specificity. The PCR results for four representative genes (*PTEN/TP53/RNF40/KMT2C*) are illustrated in Figure 3.

**Figure 4:**
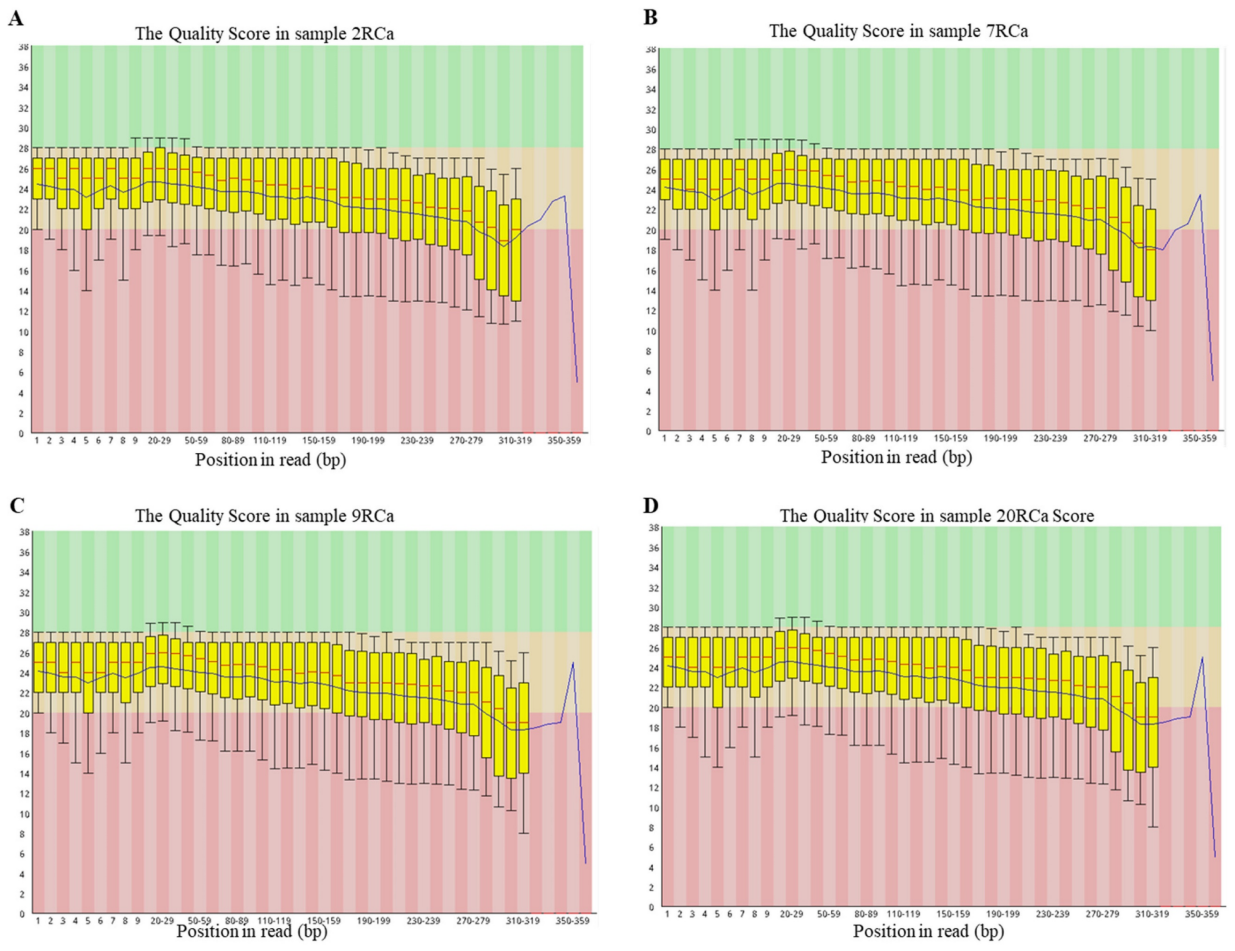
Representative quality score profiles for different samples were generated using Agilent technology. A&B&C&D. The quality score was determined for sample 2RCa, sample 7RCa, sample 9RCa, sample 20RCa, respectively.

After the successful design of the gene panel primers, MPCR amplification was conducted, and subsequent purification of the products was performed. All samples underwent library construction and successfully passed quality control measures, as depicted in Figure 4, prior to undergoing NGS. The target fragment distribution of the normal library was between 280 bp and 460 bp, and the main peak was about 391 bp. Through bioinformatics analysis of sequenced data, known various types of variants were identified for each sample [Table 3].

### 3.3 Variant verification results

**Figure 5:**
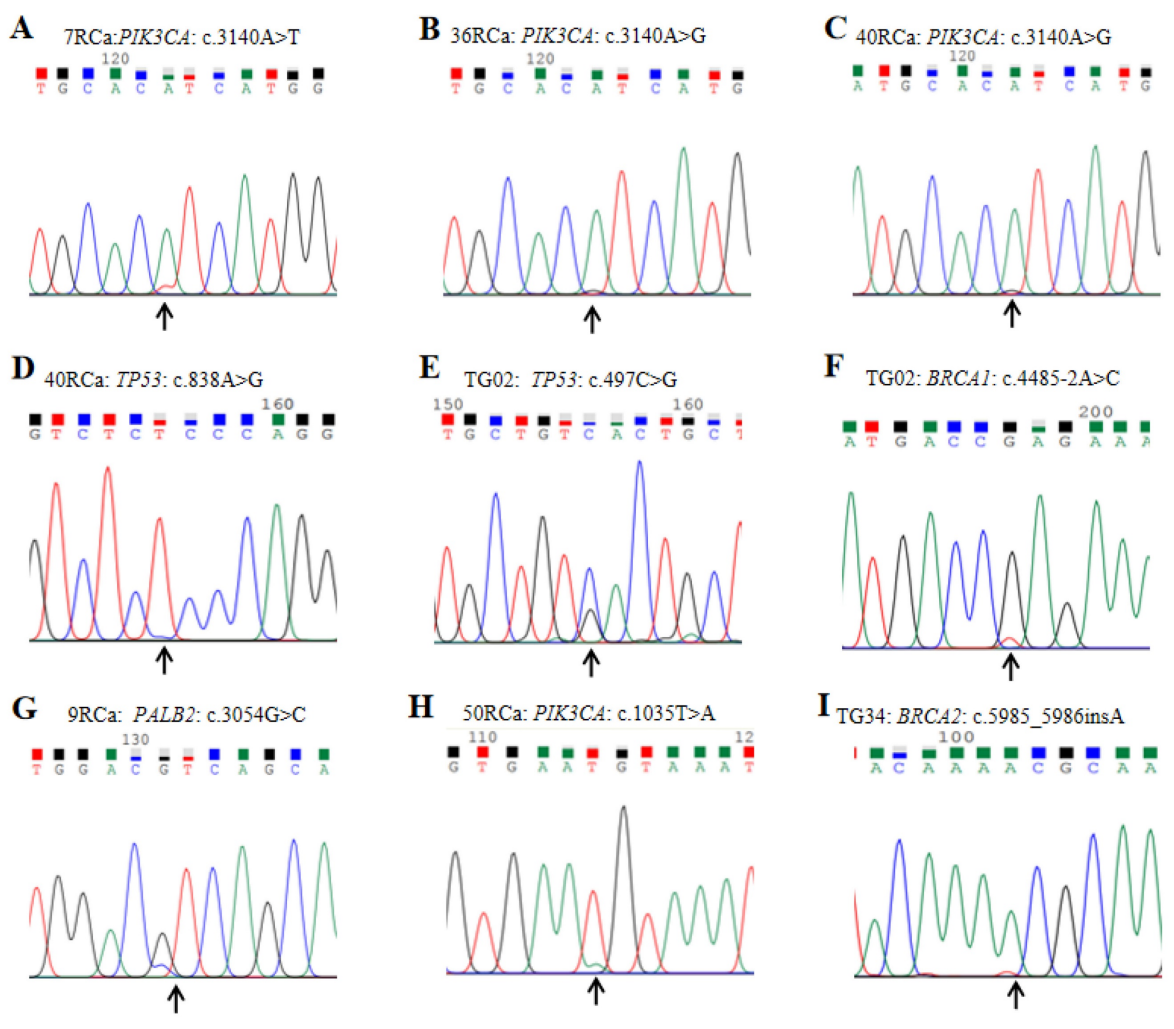
Representative program profiles for variant verification by Sanger sequencing are presented as follows: A. The Sanger sequencing results of sample 7RCa reveal the presence of *PIK3CA* variant c.3140A>T. B. In sample 36RCa, the Sanger sequencing results indicate the occurrence of *PIK3CA* variant c.3140A>G. C&D. Sample 40RCa exhibits two variants, namely *PIK3CA* variant c.3140A>T and *TP53* variant c.838A>G, as confirmed by Sanger sequencing. E&F. The Sanger sequencing analysis of sample TG02 identifies *TP53* variant c.497C>G and *BRCA1* variant c.4485-2A>C. G. The *PALB2* variant c.3054G>C is detected in sample 9RCa through Sanger sequencing. H. In sample 50RCa, a *PIK3CA* variant c.1035T>A is identified using Sanger sequencing. I. Sample TG34 shows an insertion mutation (*BRCA2* variant c.5985_5986insA) according to the results obtained from Sanger sequencing.

To validate the genetic variations identified by the gene panel, the Sanger sequencing method was employed to confirm partial variants, and representative outcomes are presented in Figure 5. All detected variants were successfully validated through Sanger sequencing. It is worth noting that certain patients exhibited pathogenic variants with SNV peak values less than one-fourth of the allele nucleotide value. Notably, different samples displayed identical variants at the same position with distinct nucleotide types, for instance, samples 7RCa and 36RCa showed *PIK3CA* variant c.3140A>T and c.3140A>G, respectively (Figure 5 A&B). Furthermore, the sequencing results revealed that a single sample harbored multiple variants in different genes; for example, sample 40RCa exhibited *PIK3CA* and *TP53* variants c.3140A>T and c.838A>G, respectively (Figure 5 C&D), while in sample TG02 demonstrated *TP53* and *BRCA1* variant c.497C>G and c.4485-2A>C, respectively (Figure 5 E&F). Additionally, we observed *PALB2* variant c.3054G>C in sample 9RCa, *PIK3CA* variant c.1035T>A in sample 50RCa, as well as *BRCA2* variant c.5985_5986insA in sample TG34, respectively (Figure 5 G&H&I).

### 3.4 Results of different variants' distribution

**Figure 6:**
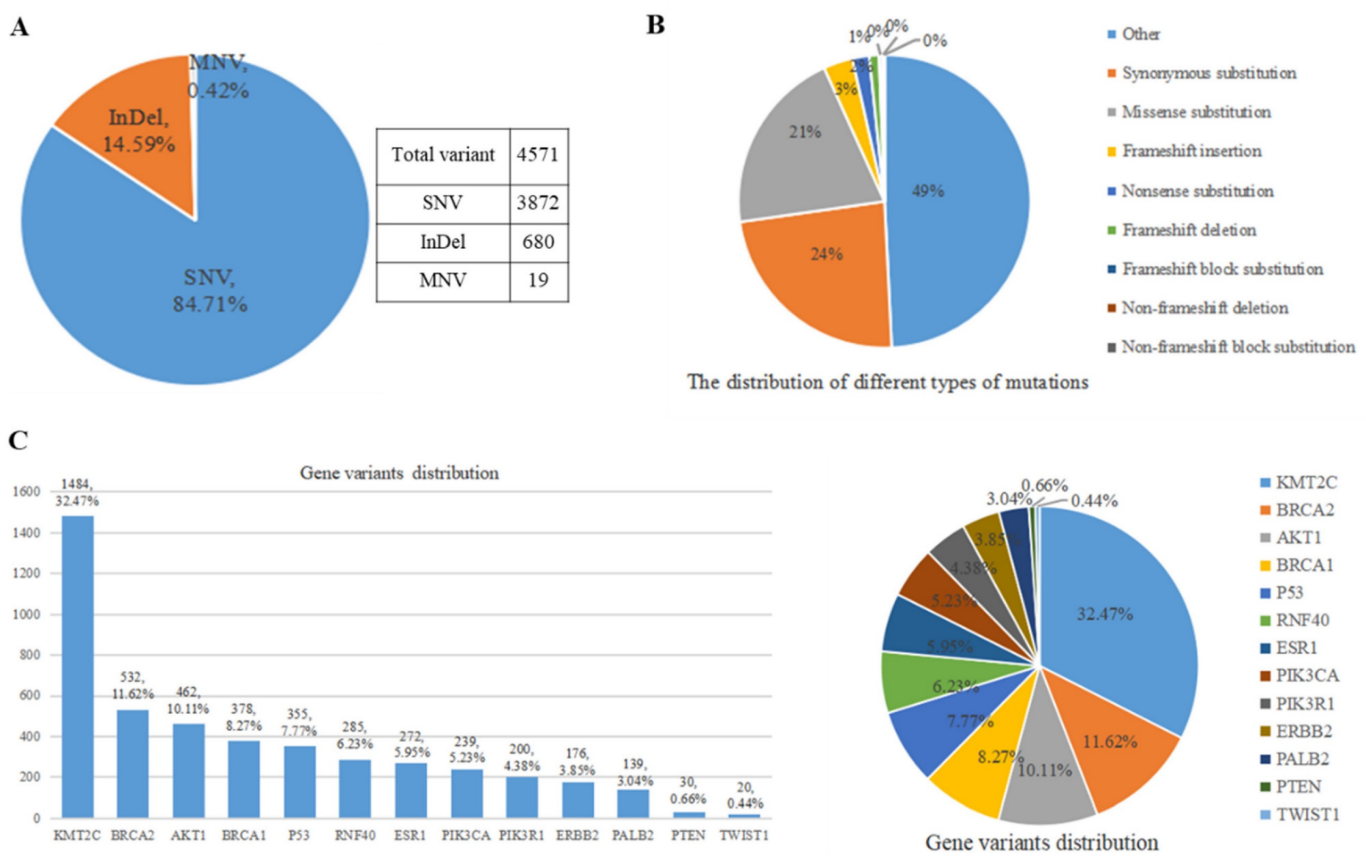
Results of different variants of the panel encompassing 13 genes examined in a cohort comprising 52 distinct breast cancer samples and individuals without the disease. A. Distribution analysis was conducted for missense, nonsense, synonymous, and intron mutation. B. The SNV, InDel, and MNV distribution was also investigated. C. Comprehensive assessment of all variant types within our gene panel was performed. Abbreviations: SNV: Single nucleotide variant; InDel: Insertion-Deletion; MNV: Multiple Nucleotide Variant.

By conducting bioinformatics analysis, 4,571 variants were identified in the annotations files from 70 samples and classified into different types (Figure 6). All the variants were somatic changes. The predominant type of variants was single nucleotide variation (SNV), accounting for approximately 84.71% (3,872 variants) of the total. The second most common type was insertion and deletion (InDel), comprising about 14.59% (680 variants) of all variants. Additionally, multiple nucleotide variants (MNV) were also observed (Figure 6A). Depending on the specific nucleotide alterations, amino acid changes may occur which can potentially impact protein function. These genetic variations were further categorized into various types, including synonymous mutations, missense mutations, frameshift insertion/deletion/block substitution, non-frameshift deletion/block substitution, and other types (Figure 6B). Figure 6B illustrates that the three most prevalent types of amino acid changes were synonymous mutations, missense mutations, and frameshift insertion with frequencies of 23.54% (1076 variants), 20.46% (935 variants), and 3.24% (148 variants), respectively. Through analyzing the distribution patterns of these variants across different genes and their positions within them (Figure 6C), it was found that* KMT2C* gene exhibited the highest number of variant occurrences with a count of around 1484 representing approximately 32.47% of all detected variants. Following this, the gene *BRCA2* displayed the second-highest frequency with 531 variants accounting for 11.62% of all detected variants. The gene *TWIST1* had the lowest number of variants with only 20 around representing approximately 0.44% of all detected variants.

### 3.5 New variants found in our cohort

**Figure 7:**
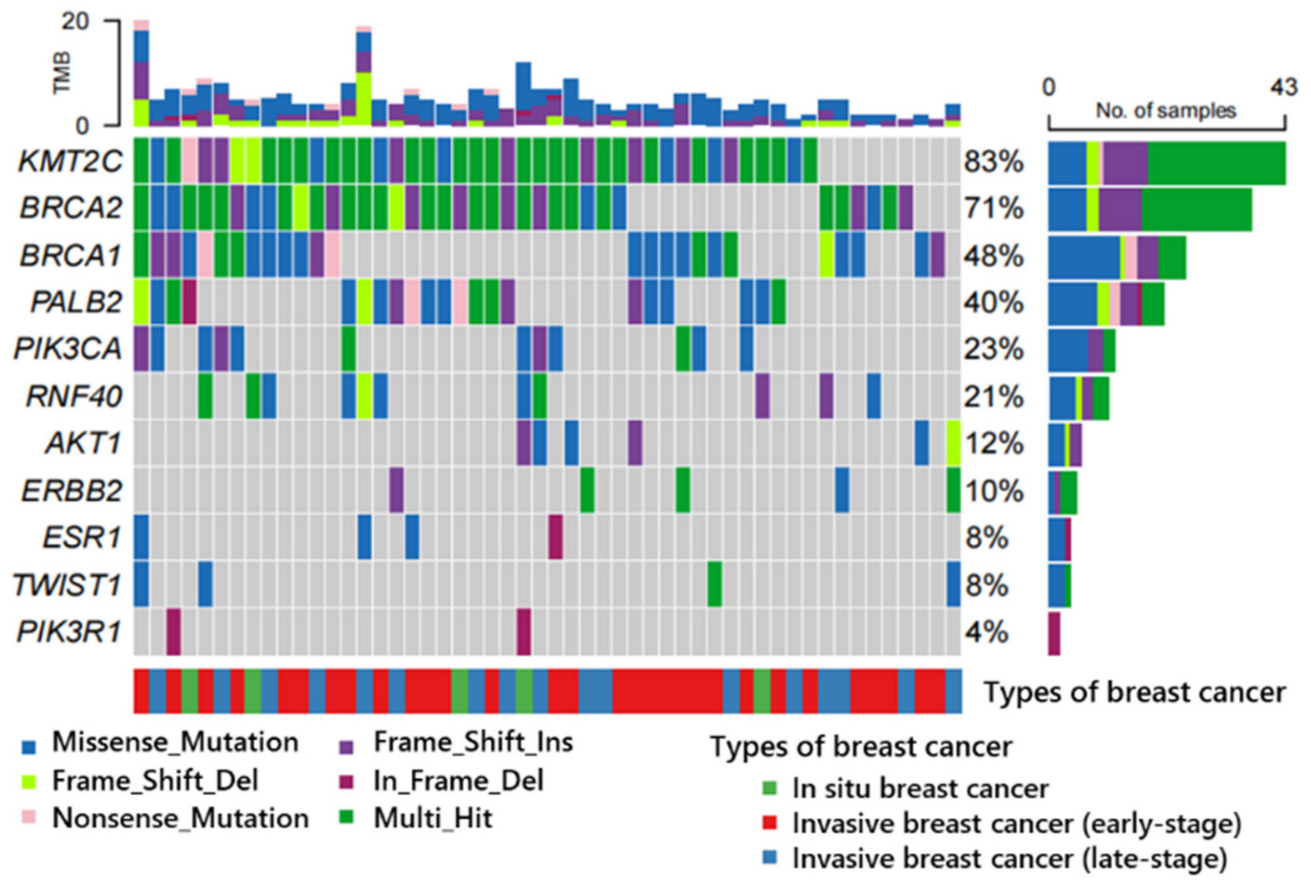
The distribution of all new variants in 11 genes (*KMT2C* (83.0%), *BRCA2*(71%), *BRCA1* (48%), *PALB2*(40%), *PIK3CA* (23%), *RNF40* (21%), *AKT1* (12%), *ERBB2* (10%), *ESR1* (8%), *TWIST1* (8%) and *PIK3R1* (4%)) was observed in a cohort of 52 patients. Notably, no new variant was identified in *TP53* and *PTEN* genes. These variants were classified into different types, including Missense_mutation, Frame_Shift_Ins, Frame_Shift_Del, Nonsense_mutation, and In_Frame_Del. Additionally, the samples were categorized into three groups: in situ breast cancer with 5 patients, invasive breast cancer (early-stage) with 30 patients, and invasive breast cancer (late-stage) with 17 patients. Abbreviations: Frame_Shift_Ins: Frameshift insertion; Frame_Shift_Del: Frame shift deletion; In_Frame_Del: Inframe deletion; Multi_Hit: Multiple Hit.

The majority of the variants were identified through comprehensive databases such as ClinVar, OMIM, COSMIC dbsnp, ExAC, etc. Variants that could not be retrieved from these databases were meticulously analyzed and considered for the first time within our cohort. The 358 novel variants (approximately 7.8% of the overall count) were discovered from 52 patients. Subsequently, bioinformatics analysis was conducted to obtain annotation files and classify these variants into distinct types (Figure 7). Figure 7 illustrates that these newly identified variants are distributed among eleven genes, with *KMT2C* gene exhibiting the highest prevalence in 83.0% of samples. Additionally,* BRCA2* (71%), *BRCA1* (48%), *PALB2* (40%), *PIK3CA* (23%), and *RNF40* (21%) genes displayed variant frequencies exceeding 20% of samples. Furthermore, *AKT1* (12%), *ERBB2* (10%), *ESR1* (8%), *TWIST1* (8%), and *PIK3R1* (4%) genes exhibited variant occurrences; however, no new variants were detected in *TP53* and *PTEN* genes.

Depending on the type of function, missense, frameshift insertion, frameshift deletion, nonsense, and in-frame deletion are observed. Missense variants constitute the predominant type with a total frequency of 40%, followed by frameshift insertion at 23%. The remaining variants collectively account for less than 40% of the occurrences. Notably, certain genes (*KMT2C* and *BRCA2*) exhibit multiple hits as previously mentioned.

We identified somatic mutations by adding blood samples of healthy individuals to exclude the germline mutations. Based on the sequencing data, the variant allele frequencies were low and all were greater than 2%, which also showed these variants were somatic mutations. PolyPhen-2 and SIFT are commonly employed in-silico tools for missense variant interpretation. A PolyPhen-2 prediction score above 0.3 indicates a probable damaging effect of the variant, resulting in a total of 89 variants (24.9%) out of 358 (52 patients) with a score exceeding this threshold value. Disease-causing (DC) variants exhibit a SIFT value below 0.05, leading to the identification of 101 variants with scores lower than this cutoff among the total number analyzed. Notably, when considering both PolyPhen-2 scores above 0.3 and the SIFT scores below 0.05, a subset of 68 pathogenic variants was found within our cohort consisting of 358 variants across 52 patients [Table 4]. At the same time, CADD and Mutation Taster tools were also used to analyze these variants. The prediction of the Mutation Taster showed that 74.24% of these variants are disease-causing (exclude 17 polymorphisms) and their corresponding probability value is shown in Table 4. In addition, 78.79% of the novel variants had a PHRED score greater than 20 in the CADD tool. Taken together, a combination of PolyPhen-2, SIFT, CADD, and Mutation Taster tools' analysis, 49 variants (49/358) had potential pathogenic effects on protein functions and structures across 52 patients. According to the silico prediction, the variants were conserved across different species and not in the germline.

## 4. Discussion

The technology developed for NGS has revolutionized the clinical approach to genetic testing in the field of cancer. Multi-gene panels have demonstrated powerful capabilities in identifying pathogenic variants (PVs) within known BC-related genes, as well as novel variants potentially associated with the disease. Recently, a combination of hereditary gene panels targeting hot mutations such as *BRCA1/2* and *PALB2,* along with multiple genes with various cancers, applied for gene diagnosis 6, 8, 12, 14, 38.

A few of these gene variants were not found in ClinVar, OMIM, and COSMIC databases within the gene panel of variants distribution. Novel mutations were initially discovered in our cohort and confirmed as novel through the ExAC database. Specifically, 68 new variants from PolyPhen-2 and SIFT analysis exhibited potential pathogenicity, providing evidence for ACMG that supports the need for more specific pathogenicity classification when combined with other evidence to enhance clinical application 47. These pathogenic variants increase the risk of BC and offer guidance for early detection/diagnosis. All newly identified variants in the *KMT2C* gene ranked the highest within our cohort, which has been recently reported as a biomarker for chemotherapy response 12, 29. The distribution pattern of new variants in *BRCA1/2* genes is as follows: *BRCA1/2* genes are associated with BC pathogenesis and account for approximately 5%-10% of known pathogenic variations among all BC-related genes; they also act as modifiers of hereditary BC risk. Analysis conducted on a large number of Chinese hereditary BC patients revealed that germline variations in the *BRCA1/2* genes exhibit high ethnic specificity 7, 14, 15, 32, 36, 37. The *PALB2* (partner and localizer of *BRCA2*) gene ranks after *KMT2C* and *BRCA1/2* genes among new variants, it encodes a protein that interacts with* BRCA2*, and its mutations are associated with significantly increased female BC risk 11. *PIK3CA* (23%) and *RNF40* (21%) variants were detected in over 20% of samples. The mutation rate of *PIK3CA* is particularly high in male breast cancer (MBC), ranking first among known BC-associated variations recorded in the COSMIC database. Among our patients' cases involving this gene mutation type p. H1047R was predominant 12, 19, 31, 51. In our novel variants of the *RNF40* gene, 19 additional variants were identified, out of which six were classified as DC mutation based on PolyPhen-2 scores greater than 0.3 and SIFT scores less than 0.05. RNF40 has been reported to exhibit both tumor-suppressive and oncogenic roles in BC cells. The occurrence of these gene variants in BC patients has been relatively infrequent 33-35. Similarly, the distribution of new variants in *AKT1* (12%), *ERBB2* (10%),* ESR1*(8%), *TWIST1*(8%), and *PIK3R1*(4%) genes was found to be less than 15% among all patients 31. These genetic variations across multiple genes pose an increased risk of BC development in humans; however, early detection can facilitate timely diagnosis, prevention, and treatment.

Variants of the *TP53* and *PTEN* genes were identified in our patient cohort. Additionally, these variants have been associated with an increased susceptibility to cancer development. Tumor protein p53 (TP53), a transcription factor, is a well-known tumor suppressor gene that exhibits high mutation rates across various cancer types, including BC. Although germline in the *TP53* gene is rare and occurs in only approximately 1% of all BC cases, carriers of such mutation face a significantly higher lifetime risk of developing BC, estimated at around 80%-90%, compared to non-carriers 14, 30. A recent study has also demonstrated that pathogenic variants in the *PTEN* gene are linked to earlier disease onset and an elevated risk for female BC 13, 14.

Despite extensive research on clinical inhibitors for most high-risk genes with hotspot variants in this gene panel 6, 10, 19-23, 25-28, 50, 55, our study acknowledges certain limitations. The majority of these variants are somatic mutations found in sporadic cases of BC. However, the resistance mechanisms to novel pathogenic variants remain elusive. Therefore, identifying and targeting these pathogenic variants for early BC treatment still presents a significant challenge.

In our study of the patient tissue with clinical information, we consistently observed a large tumor size, with a maximum diameter of 55 mm, several times larger than that seen in BC patients from Western countries. The average age at diagnosis patients was around 50 years, contrasting with the late age at diagnosis typically observed in developed Western countries. Consequently, patients with tumors were more susceptible to metastasis and recurrence, leading to a decrease in their five-year survival rate 1-3, 10. Early detection, diagnosis, and treatment of BC can improve the survival rate of patients. It is one of the few malignant tumors that can reduce the mortality rate through early diagnosis. Compared with conventional imaging examination, genetic detection has obvious advantages, which can diagnose BC earlier before the appearance of typical clinical manifestations and improve the level of cancer prevention and treatment. Therefore, the novel gene panel based on the high-risk genes may have a high value for the early detection of BC. Currently available data on germline and somatic mutations in BC patients are expanding. Our findings suggest that this novel variant holds significance for early diagnosis/prevention, and treatment strategies specifically tailored for Chinese individuals. Furthermore, it contributes to the overall knowledge base and facilitates clinical applications for diagnosing BC patients as well as providing genetic counseling services for affected families. Notably, utilizing a gene panel for patient gene diagnosis proves cost-effective compared to whole exome sequencing (WES). Compared with general NGS, the gene panel is based on high-risk genes of BC, which has fewer genes and high sequencing depth, easier interpretation, and more efficient detection. This approach not only benefits undeveloped regions of China like some areas in southwest China but also holds potential value for undeveloped countries across Asia or Africa, such as Thailand 4, 7, 10, 31, 38.

## 5. Conclusions

In conclusion, we have preliminarily established a cost-effective and high-risk gene panel for early detection of BC. We found a subset of 49 new pathogenic variants within our cohort consisting of 358 variants across 52 patients on protein functions and structures, and the non-pathogenic variants that may also play a risk factor in BC patients. This breakthrough will contribute to the timely identification, effective prevention, and preventative treatment of BC patients, particularly those residing in developing or undeveloped regions. Moreover, our cohort's discovery of novel pathogenic variants not only enriches the existing knowledge base for clinical application of genetic diagnosis in BC patients but also enhances genetic counseling services pertaining to both BC patients and their family pedigrees.

## Figures and Tables

**Table 1:** Demographic summary of the study group

	Number (n)	Percentage (%)
**Gender**		
Female	65	92.8
Male	5	7.2
**Cancer**		
Yes	52	74.3
No	18	25.7
**Age of onset**		
<40 years	13	18.6%
>40 years	57	82.4%
**Intrinsic molecular subtype of breast cancer patients**		
Luminal A	14	26.9%
Luminal B	23	44.2%
HER2- enriched	11	21.2%
Basal-like	4	7.7%
**Types of breast cancer**		
in situ breast cancer	5	9.6%
invasive breast cancer (early-stage)	30	57.7%
invasive breast cancer (late-stage)	17	32.7%

**Table 2:** List of genes analyzed and their association with breast cancer

Gene	Transcript	Chromosome	Num_primers	Num_exons	Alteration	Frequency (%)	Candidate drug	Level of evidence for the target	Gene function/signal pathway	Specific inhibitors
*PIK3CA*	NM_006218.4	chr3	43	21	Amplifications/Mutations	>10	PI3K inhibitor	1-2	PI3K/AKT/mTOR	Alpelisib (FDA-approved), Copanlisib (clinical trial)
*PIK3R1*	NM_181523.3	chr5	57	19	Mutations	1-5		4	PI3K/AKT/mTOR	
*ESR1*	NM_001122740.2	chr6	48	12	Mutations/Amplifications/Translocations	>10% in metastatic/ER+ MBC resistant to endocrine therapy		2	ER signaling	
*KMT2C*	NM_170606.3	chr7	145	59	Mutations	5-10	Drug targeting/epigenetics	4	Epigenetics	
*PTEN*	NM_000314.8	chr10	60	10	Mutations/Deletions	5-10	AKT inhibitor	3	PI3K/AKT/mTOR	Copanlisib (clinical trial)
*BRCA2*	NM_000059.4	chr13	82	27	Mutations/Deletions	1-5	PARP inhibitor	1	DNA repair	Rucaparib, Niraparib, Olaparib (FDA-approved)
*AKT1*	NM_001014431.2	chr14	30	15	Amplifications/Mutations	1-5	AKT inhibitor	2	PI3K/AKT/mTOR	Capivasertib (AZD-5363, clinical trial)
*ERBB2*	NM_004448.4	chr17	60	32	Amplifications/Mutations	>10	HER2 inhibitor	1/3	Growth factor receptors	Trastuzumab (FDA-approved)
*TP53*	NM_000546.6	chr17	24	11	Mutations	>10	Not known	NA	DNA repair	
*BRCA1*	NM_007294.4	chr17	56	24	Mutations/Deletions	1-5	PARP inhibitor	1	DNA repair	Rucaparib, Niraparib, Olaparib (FDA-approved)
*TWIST1*	NM_000474.4	chr7	11	2						
*RNF40*	NM_014771.4	chr16	48	20						
*PALB2*	NM_024675.4	chr16	32	13						

**Table 3:** Known pathogenic variants result

Sample	Type	Genotype	Gene	Function	Exon	Protein	Coding	ClinVar	Chromosome
2RCa	SNV	A/T	*PIK3CA*	missense	21	p.His1047Leu	c.3140A>T	Pathogenic	chr3:178952085
20RCa	SNV	A/G	*PIK3CA*	missense	21	p.His1047Arg	c.3140A>G	Pathogenic	chr3:178952085
26RCa	SNV	A/G	*PIK3CA*	missense	21	p.His1047Arg	c.3140A>G	Pathogenic	chr3:178952085
36RCa	SNV	CA/CG	*PIK3CA*	missense	21	p.His1047Arg	c.3140A>G	Pathogenic	chr3:178952084
40RCa	SNV	ATC/GTC	*PIK3CA*	missense	21	p.His1047Arg	c.3140A>G	Pathogenic	chr3:178952085
40RCa	SNV	T/C	*TP53*	missense	8	p.Arg280Gly	c.838A>G	Conflicting Interpretations of pathogenicity	chr17:7577100
42RCa	SNV	A/G	*PIK3CA*	missense	21	p.His1047Arg	c.3140A>G	Pathogenic	chr3:178952085
TG15	SNV	ACA/ACG	*PIK3CA*	missense	21	p.His1047Arg	c.3140A>G	Pathogenic	chr3:178952083
TG19	SNV	A/G	*PIK3CA*	missense	21	p.His1047Arg	c.3140A>G	Pathogenic	chr3:178952085
TG46	SNV	A/T	*PIK3CA*	missense	21	p.His1047Leu	c.3140A>T	Pathogenic	chr3:178952085
9RCa	SNV	C/G	*PALB2*	missense	10	p.Glu1018Asp	c.3054G>C	Conflicting Interpretations of pathogenicity	chr16:23632742
27RCa	SNV	C/T	*TP53*	nonsense	4	p.Trp53Ter	c.158G>A	Pathogenic	chr17:7579529
31RCa	INDEL	GTTTA/G	*BRCA2*	frameshiftDeletion	10	p.Ile591MetfsTer22	c.1773_1776delTTAT	Pathogenic	chr13:32907382
50RCa	SNV	T/A	*PIK3CA*	missense	5	p.Asn345Lys	c.1035T>A	Pathogenic	chr3:178921553
TG02	SNV	G/C	*TP53*	nonsense	5	p.Ser166Ter	c.497C>G	Pathogenic	chr17:7578433
TG02	SNV	T/G	*BRCA1*	unknown	14	p.?	c.4485-2A>C	Likely pathogenic	chr17:41226540
TG04	SNV	C/T	*TP53*	nonsense	4	p.Trp91Ter	c.273G>A	Pathogenic	chr17:7579414
TG34	INDEL	CAAAAC/CAAAACA	*BRCA2*	frameshiftInsertion	11	p.Ala1996SerfsTer7	c.5985_5986insA	Pathogenic	chr13:32914472
TG38	SNV	T/C	*TP53*	missense	5	p.Tyr163Cys	c.488A>G	Pathogenic	chr17:7578442
TG39	SNV	G/A	*PIK3CA*	missense	2	p.Arg38His	c.113G>A	Likely pathogenic	chr3:178916726
TG40	SNV	C/T	*TP53*	missense	7	p.Gly245Ser	c.733G>A	Pathogenic	chr17:7577548
TG52	SNV	G/C	*TP53*	missense	6	p.His193Asp	c.577C>G	Likely pathogenic	chr17:7578272

**Table 4:** Results of new probable damaging variants found in our cohort

Sample	Gene	Chromosome	Function	Exon	Protein	Coding	Variant Allele Frequency	Sift	Polyphen	MutationTaster	PHRED
2RCa	*KMT2C*	chr7:151949631	Missense_Mutation	10	p.Arg490Gly	c.1468A>G	2.94%	0	0.999	1	19.31
2RCa	*BRCA2*	chr13:32914067	Non_Synonymous_Mutation	11	p.Lys1861=;Val1862Met	c.5583_5584delAGinsGA	3.64%	0.04	0.937	poly	-
7RCa	*BRCA2*	chr13:32906985	Missense_Mutation	10	p.Lys457Arg	c.1370A>G	6.58%	0.01	0.949	poly	17.19
9RCa-1	*BRCA2*	chr13:32906459	Missense_Mutation	10	p.His282Asn	c.844C>A	4.17%	0.05	0.723	poly	6.942
10RCa	*KMT2C*	chr7:151960147	Missense_Mutation	9	p.Leu418Pro	c.1253T>C	3.51%	0	1	0.999	28.6
11RCa	*KMT2C*	chr7:151880075	Missense_Mutation	35	p.Glu1750Gly	c.5249A>G	3.57%	0	0.999	0.999	26.5
11RCa	*BRCA2*	chr13:32893237	Missense_Mutation	3	p.Trp31Arg	c.91T>A	22.49%	0	1	0.999	25.0
11RCa	*PALB2*	chr16:23619317	Missense_Mutation	12	p.Val1073Ala	c.3218T>C	3.45%	0	0.994	poly	24.2
12RCa	*KMT2C*	chr7:151853395	Missense_Mutation	45	p.Ser3903Pro	c.11707T>C	2.56%	0.02	1	0.999	26.8
12RCa	*KMT2C*	chr7:151873368	Missense_Mutation	38	p.Asp3057Gly	c.9170A>G	2.9%	0	1	0.999	26.0
12RCa	*KMT2C*	chr7:151874022	Missense_Mutation	38	p.Glu2838Val	c.8513A>T	3.13%	0	0.895	0.994	21.7
13RCa	*PALB2*	chr16:23614913	Missense_Mutation	13	p.Leu1143Pro	c.3428T>C	2.67%	0.02	0.562	0.999	24.7
16RCa	*KMT2C*	chr7:151833939	Missense_Mutation	59	p.Asn4905Ser	c.14714A>G	2.94%	0	0.997	0.999	25.9
17RCa	*TWIST1*	chr7:19156497	Missense_Mutation	1	p.Lys150Glu	c.448A>G	5.36%	0	0.993	0.999	32
17RCa	*ERBB2*	chr17:37871589	Missense_Mutation	10	p.Phe400Ser	c.1199T>C	2.7%	0	1	0.999	27.1
18RCa	*PIK3CA*	chr3:178919182	Missense_Mutation	4	p.Glu223Lys	c.667G>A	2.94%	0	0.834	0.999	25.0
18RCa	*TWIST1*	chr7:19156473	Missense_Mutation	1	p.Phe158Leu	c.472T>C	3.77%	0	1	0.999	32
18RCa	*BRCA2*	chr13:32907192	Missense_Mutation	10	p.Asp526Gly	c.1577A>G	2.88%	0.01	0.354	poly	11.21
18RCa	*RNF40*	chr16:30774446	Missense_Mutation	3	p.Met47Thr	c.140T>C	7.02%	0	0.774	0.999	25.7
25RCa	*KMT2C*	chr7:151879465	Missense_Mutation	36	p.Lys1827Arg	c.5480A>G	4.4%	0	0.999	0.868	24.3
26RCa	*KMT2C*	chr7:151873278	Missense_Mutation	38	p.Ile3087Thr	c.9260T>C	3.41%	0	0.985	0.999	29.8
26RCa	*BRCA2*	chr13:32906459	Missense_Mutation	10	p.His282Asn	c.844C>A	4.17%	0.05	0.723	poly	6.942
26RCa	*RNF40*	chr16:30774446	Missense_Mutation	3	p.Met47Thr	c.140T>C	7.02%	0	0.774	0.999	25.7
27RCa	*BRCA2*	chr13:32907224	Missense_Mutation	10	p.Glu537Gly	c.1610A>G	3.47%	0.01	0.84	poly	13.90
29RCa	*TWIST1*	chr7:19156511	Missense_Mutation	1	p.Lys145Arg	c.434A>G	2.74%	0	0.969	0.999	33
29RCa	*TWIST1*	chr7:19156527	Missense_Mutation	1	p.Ser140Thr	c.418T>A	2.74%	0	1	0.999	31
29RCa	*TWIST1*	chr7:19156545	Missense_Mutation	1	p.Ile134Val	c.400A>G	2.67%	0	0.542	0.999	30
31RCa	*BRCA1*	chr17:41245607	Missense_Mutation	10	p.Ser647Arg	c.1941T>A	3.64%	0	0.991	0.891	22.4
33RCa	*KMT2C*	chr7:151879333	Missense_Mutation	36	p.Ala1871Val	c.5612C>T	3.45%	0	0.361	poly	22.0
34RCa	*PALB2*	chr16:23614949	Missense_Mutation	13	p.Ile1131Thr	c.3392T>C	2.6%	0	0.603	0.712	25.5
36RCa	*AKT1*	chr14:105236736	Missense_Mutation	14	p.Asp462Gly	c.1385A>G	2.99%	0	0.346	0.999	19.87
36RCa	*BRCA1*	chr17:41245353	Missense_Mutation	10	p.Glu732Gly	c.2195A>G	2.67%	0	0.868	poly	13.11
40RCa	*PIK3CA*	chr3:178952118	Missense_Mutation	21	p.Ile1058Thr	c.3173T>C	2.73%	0	0.717	0.999	24.6
42RCa	*KMT2C*	chr7:151860736	Missense_Mutation	43	p.Gln3309Arg	c.9926A>G	2.74%	0.05	0.671	poly	14.94
42RCa	*PALB2*	chr16:23614925	Missense_Mutation	13	p.Ile1139Thr	c.3416T>C	2.53%	0	0.946	poly	25.1
42RCa	*PALB2*	chr16:23614931	Missense_Mutation	13	p.Ile1137Thr	c.3410T>C	3.8%	0	0.998	0.994	25.9
43RCa	*KMT2C*	chr7:151833981	Missense_Mutation	59	p.Glu4891Gly	c.14672A>G	2.7%	0	0.999	0.999	32.0
43RCa	*KMT2C*	chr7:151833997	Missense_Mutation	59	p.Tyr4886His	c.14656T>C	2.67%	0	1	0.999	29.9
43RCa	*KMT2C*	chr7:151876970	Missense_Mutation	37	p.Gly2464Glu	c.7391G>A	2.82%	0.01	0.998	0.999	24.8
43RCa	*BRCA2*	chr13:32910755	Missense_Mutation	11	p.Ser755Pro	c.2263T>C	2.96%	0.03	0.469	poly	11.00
43RCa	*RNF40*	chr16:30780523	Missense_Mutation	16	p.Leu755Pro	c.2264T>C	2.52%	0	1	0.999	29.6
46RCa	*BRCA2*	chr13:32912838	Missense_Mutation	11	p.Phe1449Ser	c.4346T>C	2.56%	0	0.861	poly	22.7
46RCa	*PALB2*	chr16:23619314	Missense_Mutation	12	p.Leu1074Arg	c.3221T>G	4.11%	0	1	0.986	27.1
46RCa	*PALB2*	chr16:23619329	Missense_Mutation	12	p.Leu1069Arg	c.3206T>G	2.78%	0	1	0.999	26.1
55RCa	*RNF40*	chr16:30776578	Missense_Mutation	7	p.Gln283Arg	c.848A>G	4.84%	0	0.999	0.999	24.0
TG23	*ERBB2*	chr17:37873643	Missense_Mutation	15	p.Gly603Asp	c.1808G>A	2.7%	0	1	0.999	28.9
TG30	*KMT2C*	chr7:151849828	Missense_Mutation	49	p.Leu4163Pro	c.12488T>C	3.85%	0	0.998	0.999	24.6
TG30	*BRCA2*	chr13:32911820	Missense_Mutation	11	p.Glu1110Lys	c.3328G>A	5.11%	0	1	0.999	27.4
TG30	*PALB2*	chr16:23625332	Missense_Mutation	11	p.Ser1065Phe	c.3194C>T	3.57%	0	1	0.999	28.9
TG01	*BRCA2*	chr13:32937375	Missense_Mutation	18	p.Asp2679Gly	c.8036A>G	2.9%	0	0.997	0.999	26.0
TG02	*BRCA1*	chr17:41245161	Missense_Mutation	10	p.Thr796Ile	c.2387C>T	15.76%	0.01	0.707	poly	15.36
TG04	*BRCA2*	chr13:32971060	Missense_Mutation	26	p.Ala3176Val	c.9527C>T	3.7%	0	0.989	0.995	23.0
TG15	*BRCA2*	chr13:32906526	Missense_Mutation	10	p.Glu304Gly	c.911A>G	3.33%	0.05	0.996	poly	22.9
TG19	*KMT2C*	chr7:152008963	Missense_Mutation	5	p.Gln220Arg	c.659A>G	3.7%	0	0.534	poly	18.14
TG26	*PALB2*	chr16:23614919	Missense_Mutation	13	p.Asp1141Gly	c.3422A>G	3.08%	0.01	1	0.999	26.7
TG26	*BRCA1*	chr17:41243655	Missense_Mutation	10	p.Ser1298Pro	c.3892T>C	2.67%	0	0.998	0.975	23.7
TG38	*AKT1*	chr14:105239701	Missense_Mutation	10	p.Leu282Val	c.844C>G	26.9%	0	0.464	0.999	23.2
TG38	*RNF40*	chr16:30779233	Missense_Mutation	12	p.Met483Thr	c.1448T>C	3.77%	0	0.999	0.999	27.8
TG39	*PIK3CA*	chr3:178938941	Missense_Mutation	14	p.Gln728Arg	c.2183A>G	3.31	0	0.985	0.999	23.6
TG40	*PALB2*	chr16:23614982	Missense_Mutation	13	p.Glu1120Gly	c.3359A>G	3.66%	0	1	0.999	25.8
TG40	*RNF40*	chr16:30774446	Missense_Mutation	3	p.Met47Thr	c.140T>C	7.02%	0	0.774	0.999	25.7
TG52	*KMT2C*	chr7:151949652	Missense_Mutation	10	p.Leu483Pro	c.1448T>C	2.94%	0	1	0.999	27.0
60RCa	*PIK3CA*	chr3:178928058	Missense_Mutation	8	p.Trp446Arg	c.1336T>A	3.17%	0	1	0.999	28.2
60RCa	*KMT2C*	chr7:151845181	Missense_Mutation	52	p.Phe4611Leu	c.13831T>C	3.09%	0	0.999	0.999	23.7
60RCa	*KMT2C*	chr7:151845729	Missense_Mutation	52	p.Val4428Ala	c.13283T>C	2.55%	0.01	0.944	0.999	26.6
60RCa	*BRCA1*	chr17:41244886	Missense_Mutation	10	p.His888Tyr	c.2662C>T	3.13%	0.02	0.767	poly	12.67

Note: Here, 49 variants had potential pathogenic effects on protein functions and structures across 52 patients. poly: polymorphism
